# Effect of Parental Severe Mental Disorders on the Timing of Autism Diagnosis: A Family Linkage Study

**DOI:** 10.1007/s10803-024-06518-9

**Published:** 2024-08-13

**Authors:** Yu Tuan, Li-Chi Chen, I.-Chun Chen, Shih-Jen Tsai, Tzeng-Ji Chen, Mu-Hong Chen

**Affiliations:** 1 Department of Psychiatry, Taichung Veterans General Hospital, Taichung, Taiwan; 2 Rong Hsing Research Center for Translational Medicine, National Chung Hsing University, Taichung, Taiwan; 3 Department of Psychiatry, Taipei Veterans General Hospital, Taipei, Taiwan; 4 Department of Psychiatry, College of Medicine, National Yang Ming Chiao Tung University, Taipei, Taiwan; 5 Department of Family Medicine, Taipei Veterans General Hospital, Taipei, Taiwan; 6 Institute of Hospital and Health Care Administration, National Yang Ming Chiao Tung University, Taipei, Taiwan; 7 Department of Psychiatry, General Cheng Hsin Hospital, Taipei, Taiwan; 8 Department of Family Medicine, Taipei Veterans General Hospital, Hsinchu Branch, Hsinchu, Taiwan; 9 Department of Medical Research, Taipei Veterans General Hospital, No. 201, Sec. 2, Shih-Pai Road, Taipei, 112 Taiwan

**Keywords:** Autism, Age of diagnosis, Parents, Schizophrenia, Substance use disorder, Bipolar disorder

## Abstract

The mean diagnosis age of autism was about 5 years in Taiwan. Whether the delayed diagnosis of autism (≥ 6 years) was associated with parental severe mental disorders remained unknown. The parents of 22,859 autistic individuals and 228,590 age- and sex-matched nonautistic individuals were assessed for the presence of severe mental disorders (schizophrenia, bipolar disorder, major depressive disorder, alcohol use disorder, and substance use disorder). The timing of autism diagnosis was classified into three age categories: < 6 years, 6–11 years, and ≥ 12 years. Logistic regression models were used to examine associations between parental severe mental disorders and these age categories of autism diagnosis. Parental schizophrenia and substance use disorders were associated with the delayed diagnosis of autism, both diagnosis at ≥ 12 years (odds ratio [OR]: 2.14; 1.57) and at 6–11 years (1.87; 1.38). Parental bipolar disorder and major depressive disorder were also associated with the delayed diagnosis of autism, especially diagnosis at 6–11 years (OR 1.98; 1.86). Our findings underscore the need for clinicians to monitor the neurodevelopmental conditions of offspring born to parents with severe mental disorders during the early stages of their life.

## Introduction

Autism is a common neurodevelopmental condition characterized mainly by social impairments, deficits in both language and nonverbal communication abilities, and repetitive, fixed interests or behaviors (Lord et al., 2018). The prevalence of autism ranges from approximately 0.6–1.0% of the general population in the United States and Europe, with a male-to-female ratio ranging from 3:1 to 4:1 (Solmi et al., 2022; Talantseva et al., 2023). Reports from the World Health Organization indicate a clear increase in the prevalence (39.3%) and Disability-Adjusted Life Year (38.7%) of autism between 1990 and 2019 (Li et al., 2022).

Studies have increasingly reported a coaggregation of autism, schizophrenia, bipolar disorder, major depressive disorder, and substance and alcohol use disorders within families (Daniels et al., 2008; Davis et al., 1992; Wang et al., 2022). Shayestehfar et al. revealed that parents of autistic children were more likely to receive a diagnosis of schizophrenia compared with those of nonautistic children (Shayestehfar et al., 2023). Davis et al. and Daniels et al. discovered an association between parental substance and alcohol use disorders, such as cocaine use disorder, and the likelihood of offspring autism (Daniels et al., 2008; Davis et al., 1992). Our previous study indicated that both paternal and maternal depression were significantly associated with the likelihood of autism in the children (Chen et al., 2020). Furthermore, Sipsock et al. discovered that the severity of autism was associated with cumulative numbers of major psychiatric disorders, including schizophrenia, major affective disorders, and substance and alcohol use disorders, of their first-degree relatives (Sipsock et al., 2021).

The early identification and diagnosis of autism, ideally between 18 and 24 months of age, is crucial for enhancing the overall outcomes in autistic children. Despite the ability of experienced physicians to detect autism in children before the age of 2 years, delayed diagnoses are prevalent, particularly in autistic children who exhibit normal verbal skills (Feng-lei Zhu et al., 2023). A meta-analysis of studies from 40 countries revealed that the age at which autism was diagnosed varied widely, spanning from 38 to 120 months, with an average of approximately 60 months (van’t Hof et al., 2021). Wei et al. reported that the mean age of autism diagnosis was approximately 5.07 ± 3.10 years in Taiwan (Wei et al., 2021). Leng et al. discovered that up to 16% of autistic children were diagnosed after starting school, and also suggested that children from immigrant families commonly received delayed diagnosis (Leng et al., 2023). Durkin et al. highlighted that race and socioeconomic background can influence the age at which autism is diagnosed in the United States (Durkin et al., 2017). These studies suggest that the variance in the age of autism diagnosis reflects the biopsychosocial nature of autism.

Data are scarce on the relationship between the age of autism diagnosis in children and severe mental disorders of their parents. This study aimed to investigate the association between severe parental mental disorders, namely schizophrenia, bipolar disorder, major depressive disorder, and substance and alcohol use disorders, and the age of autism diagnosis using data from the Taiwan National Health Insurance Research Database. Given evidence that the average diagnosis age of autism ranges between 2 and 5 years (Levy et al., 2009; Wei et al., 2021), we hypothesized that parental severe mental disorders may be associated with the delayed diagnosis (≥ 6 years) of autism.

## Methods

### Data Source

The Taiwan National Health Insurance Research Database (NHIRD), which comprises healthcare data from > 99.7% of Taiwan’s population, was audited and released by the Taiwan National Health Research Institute for scientific research upon a formal application. The insurance claim information of the subjects is anonymous to maintain privacy. Comprehensive information on insured subjects is included in the database, such as demographic data, clinical visit dates, disease diagnoses, and prescriptions, between 2000 and 2011. The diagnostic codes used were based on the International Classification of Diseases, 9th Revision, Clinical Modification (ICD-9-CM). The National Health Insurance Research Database has been extensively used in many Taiwanese epidemiologic studies (; Chen et al., 2016; Zhang et al., 2021). The Institutional Review Board of our Hospital reviewed and approved this study protocol.

### Inclusion Criteria for Autistic Individuals

The autistic group was defined as autistic people (single offspring) born between 1990 and 2010 who had received an autism diagnosis (ICD-9-CM codes: 299.0, 299.8, and 299.9) from board-certified psychiatrists at least twice. Non-autistic individuals were paired in a ratio of 10:1 with autistic individuals based on their birth year, sex, family income, and level of urbanization after those who had an autism diagnosis (ICD-9-CM code: 299) anytime in the database were excluded. Autistic individuals were categorized into three subgroups based on age at autism diagnosis: < 6 years, 6–11 years, and ≥ 12 years. Parental severe mental disorders were assessed between autistic and non-autistic individuals. Severe mental disorders include schizophrenia, bipolar disorder, major depressive disorder, alcohol use disorder, and substance use disorder. As a proxy for healthcare accessibility in Taiwan, income level (levels 1–3 per month: < 19,100 new Taiwan dollars [NTD], 19,100 ~ 42,000 NTD, and > 42,000 NTD) and urbanization level of residence (levels 1–5, most to least urbanized) were evaluated (Liu et al., 2006).

### Statistical Analysis

For between-group comparisons, the independent *t*-test was used for continuous variables, and Pearson’s χ2 test was used for nominal variables. Logistic regression models with adjustments for birth-year, sex, family income, and level of urbanization were performed to investigate the association between age at autism diagnosis (< 6 years, 6–11 years, and ≥ 12 years) and parental severe mental disorders (schizophrenia, bipolar disorder, major depressive disorder, alcohol use disorder, and substance use disorder). We further assessed the effects of age at autism diagnosis on the likelihoods of paternal and maternal psychiatric disorders, separately. Finally, because current findings support greater growth in language and adaptive skills when children are identified by age 4, we additionally assessed associations between parental severe mental disorders and autism diagnosis age < 4 years, as well as autism diagnosis age 4–5 years. SAS 9.2 (SAS Institute, Cary, NC, USA) was used for all statistical analyses. All tests were two-tailed, and *p* < 0.05 was considered statistically significant.

### Community Involvement

The Taiwan NHIRD comprises healthcare data from > 99.7% of Taiwan’s population, including autistic individuals. The medical records of autistic individuals who sought medical consultation and help were included in the NHIRD. The insurance claim information of autistic and nonautistic individuals is anonymous to maintain privacy.

## Results

In all, 22,859 autistic individuals and 228,590 non-autistic individuals were enrolled in the present study, with a mean age of 12.43 ± 4.62 years and a male predominance (84.6%) (Table 1). More than half of autistic individuals (51.2%) were diagnosed in preschool (< 6 years); 34.4% at the age of 6–11 years; and 14.4% at the age of ≥ 12 years (Table 1). Parents of autistic individuals were more likely to be diagnosed with any severe mental disorder, including schizophrenia (1.1 vs. 0.6%, *p* < 0.001), bipolar disorder (0.9 vs. 0.6%, *p* < 0.001), major depressive disorder (8.0 vs. 4.9%, *p* < 0.001), and substance use disorder (1.6 vs. 1.3%, *p* = 0.002), than did parents of non-autistic individuals (Table 1). The prevalence of alcohol use disorder did not differ (*p* = 0.722) between parents of autistic and non-autistic individuals (Table 1).

**Table 1:** Demographic characteristics between autistic and non-autistic individuals

	Autistic individuals (*n* = 22,859)	Non-autistic individuals (*n* = 228,590)	
Age at study end (years, SD)	12.43 (4.62)	12.43 (4.62)	0.978
Age at autism diagnosis (SD, n, %)	6.86 (4.03)		
< 6 years	11,694 (51.2)		
< 4 years	6970 (30.5)		
4–5 years	47.24 (20.7)		
6–11 years	7873 (34.4)		
≥ 12 years	3296 (14.4)		
Sex (n, %)			> 0.999
Female	3531 (15.4)	35,310 (15.4)	
Male	19,328 (84.6)	193,280 (84.6)	
Parental mental disorder (n, %)			
Schizophrenia	253 (1.1)	1448 (0.6)	< 0.001
Bipolar disorder	213 (0.9)	1349 (0.6)	< 0.001
Major depressive disorder	1822 (8.0)	111,93 (4.9)	< 0.001
Substance use disorder	366 (1.6)	3080 (1.3)	0.002
Alcohol use disorder	317 (1.4)	3107 (1.4)	0.722
Paternal mental disorder (n, %)			
Schizophrenia	129 (0.6)	786 (0.3)	< 0.001
Bipolar disorder	81 (0.4)	569 (0.2)	0.003
Major depressive disorder	651 (2.8)	4501 (2.0)	< 0.001
Substance use disorder	241 (1.1)	2061 (0.9)	0.023
Alcohol use disorder	227 (1.0)	2239 (1.0)	0.836
Maternal mental disorder (*n*, %)			
Schizophrenia	132 (0.6)	669 (0.3)	< 0.001
Bipolar disorder	133 (0.6)	797 (0.3)	< 0.001
Major depressive disorder	1238 (5.4)	7077 (3.1)	< 0.001
Substance use disorder	132 (0.6)	1064 (0.5)	0.022
Alcohol use disorder	93 (0.4)	893 (0.4)	0.703
Level of urbanization (n, %)			> 0.999
1 (most urbanized)	4228 (18.5)	42,280 (18.5)	
2	7080 (31.0)	70,800 (31.0)	
3	1660 (7.3)	16,600 (7.3)	
4	1517 (6.6)	15,170 (6.6)	
5 (most rural)	8374 (36.6)	83,740 (36.6)	
Income-related insured amount (n, %)			> 0.999
< 19,100 NTD/month	4121 (18.1)	41,210 (18.1)	
19,100 ~ 42,000 NTD/month	7825 (34.2)	78,250 (34.2)	
> 42,000 NTD/month	10,913 (47.7)	109,130 (47.7)	

*NTD* new Taiwan dollar

There were substantial variations in the odds ratios (ORs) for parental schizophrenia, bipolar disorder, and major depressive disorder among autistic children aged < 6 years, those aged 6–11 years, and those aged ≥ 12 years (Table 2). ORs in parental diagnoses of schizophrenia (2.14, 95% CI 1.62–2.82) and substance use disorder (1.57, 1.24–1.99) were highest in the age at autism diagnosis ≥ 12 years group (Table 2). The highest likelihoods of parental bipolar disorder (OR 1.98, 95% CI 1.61–2.44) and major depression (1.86, 1.72–2.02) were noted in the age at autism diagnosis 6–11 years group (Table 2). The risk of parental alcohol use disorder did not differ between the three autism groups after adjusting for demographic data (Table 2).

**Table 2:** Risks of parental severe mental disorders between autistic and non-autistic individuals

	Parental severe mental disorders (OR, 95% CI)				
	Schizophrenia	Bipolar disorder	Major depressive disorder	Substance use disorder	Alcohol use disorder
	Any parent				
Age at autism diagnosis					
< 6 years	**1.51 (1.23–1.86)**	**1.26 (1.00–1.59)**	**1.54 (1.43–1.66)**	0.94 (0.80–1.12)	0.89 (0.74–1.06)
< 4 years	**1.50 (1.15–1.97)**	1.17 (0.85–1.60)	**1.57 (1.42–1.73)**	0.87 (0.70–1.0)	0.99 (0.79–1.23)
4–5 years	**1.53 (1.12–2.08)**	1.39 (1.00–1.94)	**1.50 (1.34–1.69)**	1.05 (0.82–1.34)	0.75 (0.56–1.01)
6–11 years	**1.87 (1.53–2.30)**	**1.98 (1.61–2.44)**	**1.86 (1.72–2.02)**	**1.38 (1.17–1.63)**	1.14 (0.95–1.36)
≥ 12 years	**2.14 (1.62–2.82)**	**1.58 (1.14–2.20)**	**1.71 (1.52–1.93)**	**1.57 (1.24–1.99)**	1.13 (0.87–1.46)

*OR* odds ratio, *CI* confidence interval

Adjusted by demographic characteristics

**Bold** type indicates statistical significance

**Fig. 1:**
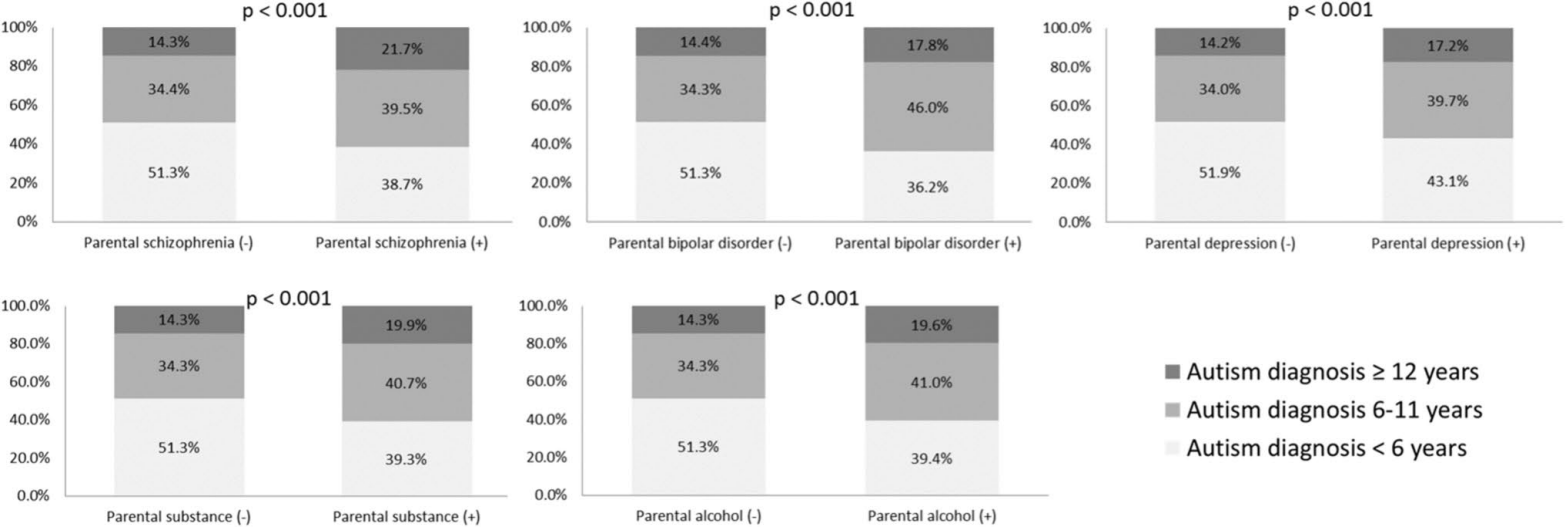
Parental severe mental disorders on the distribution of autism diagnosis (*n* = 22,859)

The subanalyses stratified by fathers and mothers showed consistent findings of associations between parental severe mental disorders and age at autism diagnosis (Table 3). Specifically, ORs in paternal schizophrenia (2.15, 95% CI 1.44–3.21), maternal schizophrenia (2.27, 1.58–3.27), and maternal substance use disorder (2.10, 1.48–2.98) were highest in the age at autism diagnosis ≥ 12 years group (Table 3). The likelihoods of maternal bipolar disorder (OR 2.20, 95% CI 1.70–2.84) and major depression (2.06, 1.89–2.27) were highest in the age at autism diagnosis 6–11 years group (Table 3). Finally, Fig. 1 showed the distribution of the age of autism diagnosis based on the presence vs. absence of specific parental severe mental disorder. Finally, sub-analyses stratified by autism diagnosis age < 4 years and 4–5 years showed similar risks of parental severe mental disorders between the two subgroups (Tables 2 and 3).

**Table 3:** Risks of paternal or maternal severe mental disorders between autistic and non-autistic individuals

	Parental severe mental disorders (OR, 95% CI)				
	Schizophrenia	Bipolar disorder	Major depressive disorder	Substance use disorder	Alcohol use disorder
	Fathers				
Age at autism diagnosis					
< 6 years	**1.41 (1.06–1.86)**	1.13 (0.78–1.64)	**1.48 (1.32–1.67)**	1.04 (0.85–1.27)	0.93 (0.75–1.14)
< 4 years	1.32 (0.91–1.91)	0.95 (0.56–1.63)	**1.49 (1.27–1.73)**	0.96 (0.74–1.25)	1.08 (0.83–1.39)
4–5 years	**1.53 (1.02–2.30)**	1.38 (0.83–2.31)	**1.48 (1.24–1.76)**	1.16 (0.87–1.54)	0.73 (0.51–1.03)
6–11 years	**1.76 (1.31–2.35)**	**1.67 (1.18–2.35)**	**1.48 (1.30–1.69)**	**1.29 (1.04–1.59)**	1.10 (0.89–1.36)
≥ 12 years	**2.15 (1.44–3.21)**	**1.66 (1.01–2.71)**	**1.37 (1.12–1.67)**	1.34 (0.98–1.83)	1.06 (0.78–1.45)

	Mothers				
Age at autism diagnosis					
< 6 years	**1.76 (1.30–2.38)**	1.33 (0.99–1.78)	**1.56 (1.42–1.71)**	0.76 (0.55–1.05)	0.79 (0.56–1.23)
< 4 years	**1.89 (1.29–2.79)**	1.29 (0.88–1.91)	**1.55 (1.37–1.75)**	0.71 (0.46–1.08)	0.78 ((0.50–1.24)
4–5 years	**1.60 (1.01–2.54)**	1.37 (0.88–2.11)	**1.57 (1.35–1.80)**	0.84 (0.54–1.34)	0.80 (0.47–1.37)
6–11 years	**2.05 (1.55–2.72)**	**2.20 (1.70–2.84)**	**2.06 (1.89–2.27)**	**1.55 (1.19–2.02)**	1.23 (0.89–1.69)
≥ 12 years	**2.27 (1.58–3.27)**	1.49 (0.96–2.32)	**1.89 (1.64–2.17)**	**2.10 (1.48–2.98)**	1.33 (0.85–2.09)

OR odds ratio; CI: confidence interval

Adjusted by demographic characteristics

**Bold** type indicates statistical significance

## Discussion

Our findings partially supported our hypothesis that parental severe mental disorders, namely schizophrenia, bipolar disorder, major depressive disorder, and substance use disorder, were associated with the delayed diagnosis (≥ 6 years) of autism. Furthermore, given that autism was diagnosed in children < 6 years old in most cases, parental schizophrenia and substance use disorder were associated with the most delayed diagnosis of autism (≥ 12 years), whereas parental bipolar disorder and major depression were associated with a less delayed diagnosis of autism (6–11 years).

Diagnosing autism requires experienced practitioners and thorough observation of various signs by primary caregivers (Levy et al., 2009). Amin et al. found more profound autistic symptoms, measured by the parent-reported Autism Quotient-Child questionnaire, in children of parents with severe mental disorders (i.e., schizophrenia) than did those of parents without severe mental disorders (Amin & Salah EL-Deen, 2021), which may logically lead to an early identification of autism. However, we discovered that parental severe mental disorders, especially schizophrenia and substance use disorders, were associated with a later diagnosis of autism in the offspring. We speculate that the compromised mental health of the parents made it more difficult for them to learn about autism and other neurodevelopmental issues and to closely monitor the neurodevelopment of their children. Evidence has shown that compared with healthy parents, parents with severe mental disorders, such as schizophrenia and major depressive disorder, exhibit poorer parenting skills, including active involvement, monitoring, and supervision (Healy et al., 2016; Kahng et al., 2008; Oyserman et al., 2000; Rabha et al., 2021; Riordan et al., 1999). Goodman et al. revealed that parental behaviors toward their children (mean age 2 years) in the schizophrenia group were associated with reduced responsiveness and stimulation, less affectionate involvement, and a poorer child-rearing environment compared with behaviors in the control group (Goodman, 1987). Kahng et al. demonstrated that the nurturance exhibited by parents with severe mental disorders improved as their symptoms became less severe (Kahng et al., 2008). The hypothesis that parents’ overall and cognitive function may affect the timing of autism diagnosis in their children and the evidence that patients with schizophrenia and substance use disorder possess more functional deficits than those with bipolar disorder and major depression (Eslami Shahrbabaki et al., 2022; Ramey & Regier, 2019; Zhu et al., 2019) may explain our findings that delayed diagnosis of autism was mostly associated with parental schizophrenia and substance use disorder, followed by parental bipolar disorder and major depressive disorder. However, further clinical studies would be required to validate the above hypothesis of the association between parental severe mental disorders and delayed autism diagnosis.

A group of autistic children being raised by parents with severe mental illness are also at risk for co-occurring mental health challenges. Evidence has shown the common mental comorbidities, such as attention deficit hyperactivity disorder (ADHD), anxiety disorders, and mood disorders, with autism, which may also be related to the delayed diagnosis of autism (Gunnarsdottir et al., 2018; Karunakaran et al., 2020; Wei et al., 2021). Our previous study assessed the sequence of autism and ADHD diagnoses and found that ADHD comorbidity may delay autism identification in autistic children with ADHD (Wei et al., 2021). Emotional and behavioral disturbances stemming from the comorbidities may mask or overlap the symptoms of autism, resulting in a delayed diagnosis (Gunnarsdottir et al., 2018; Karunakaran et al., 2020).

This study has several limitations. First, in Taiwan, the NHIRD provides highly affordable and inexpensive medical services to the public. People can directly access the specialists, including psychiatrists, without the need for a referring note. However, the prevalence of autism and parental severe mental disorders may be still underestimated because only those who sought medical and mental health consultations would be included in the database. In addition, the present study used the ICD-9-CM codes for the identification of autism and severe mental disorders. The lack of a semi-structured interview to evaluate psychopathology likely led to the omission of some mental disorders, including autism. However, the severe mental disorders in parents and autism in offspring were diagnosed by board-certified psychiatrists, which enhances diagnostic validity. Second, our study was a cross-sectional study, which limited our ability to clarify the temporality between parental severe mental disorder diagnoses and offspring autism diagnoses. One possibility was that the mental diagnoses given to the parents after the birth of their autistic children may suggest that the parents’ mental disorders, such as depression, are a consequence of raising an autistic child (Picardi et al., 2018). Chien et al. reported that parental severe mental disorders both before children’s birth and after children’s birth were associated with offspring autism risk (Chien et al., 2022).

To clarify the aforementioned temporality, further clinical longitudinal studies would be required. Fourth, the database failed to provide some information, including information on disease severity, environmental factors, and cultural factors. Therefore, we could not examine the effects of these factors in the present study. Finally, further investigation would be needed to verify whether our findings can extend to people in Western countries because social attitudes toward diagnosing autism differs between Taiwan and Western countries (Babik & Gardner, 2021).

In conclusion, our study established that delayed diagnosis of autism was mostly associated with parental schizophrenia and substance use disorder, followed by parental major affective disorders. These findings inform clinicians and public health officers about the importance of early monitoring of neurodevelopmental conditions among the offspring of parents with severe mental disorders. Compared with cases of delayed diagnoses, the timely detection of such conditions enables prompt treatment with tailored interventions as well as the full utilization of public support systems, leading to superior overall outcomes for children.

## Data Availability

Upon request, the corresponding author will provide the data supporting the study’s conclusions. The Taiwanese ethical regulations prevent the data from being made publicly available.

## References

[CR1] Amin, S. I., & Salah EL-Deen, G. M. (2021). Autistic traits in offspring of schizophrenic patients in comparison to those of normal population. *Middle East Curr Psychiatry*. 10.1186/s43045-021-00100-0

[CR2] Babik, I., & Gardner, E. S. (2021). Factors affecting the perception of disability: A developmental perspective. *Frontiers in Psychology,**12*, 702166. 34234730 10.3389/fpsyg.2021.702166 PMC8255380

[CR3] Chen, L. C., Chen, M. H., Hsu, J. W., Huang, K. L., Bai, Y. M., Chen, T. J., Wang, P. W., Pan, T. L., & Su, T. P. (2020). Association of parental depression with offspring attention deficit hyperactivity disorder and autism spectrum disorder: A nationwide birth cohort study. *Journal of Affective Disorders,**277*, 109–114. 32805586 10.1016/j.jad.2020.07.059

[CR4] Chen, M. H., Hsu, J. W., Huang, K. L., Bai, Y. M., Ko, N. Y., Su, T. P., Li, C. T., Lin, W. C., Tsai, S. J., Pan, T. L., Chang, W. H., & Chen, T. J. (2018a). Sexually transmitted infection among adolescents and young adults with attention-deficit/hyperactivity disorder: A nationwide longitudinal study. *Journal of the American Academy of Child and Adolescent Psychiatry,**57*(1), 48–53. 29301669 10.1016/j.jaac.2017.09.438

[CR5] Chen, M. H., Hsu, J. W., Huang, K. L., Su, T. P., Li, C. T., Lin, W. C., Tsai, S. J., Cheng, C. M., Chang, W. H., Pan, T. L., Chen, T. J., & Bai, Y. M. (2018b). Risk and coaggregation of major psychiatric disorders among first-degree relatives of patients with bipolar disorder: A nationwide population-based study. *Psychological Medicine,**49*, 1–8. 30415649 10.1017/S003329171800332X

[CR6] Chen, M. H., Lan, W. H., Hsu, J. W., Huang, K. L., Su, T. P., Li, C. T., Lin, W. C., Tsai, C. F., Tsai, S. J., Lee, Y. C., Chen, Y. S., Pan, T. L., Chang, W. H., Chen, T. J., & Bai, Y. M. (2016). Risk of developing type 2 diabetes in adolescents and young adults with autism spectrum disorder: A nationwide longitudinal study. *Diabetes Care,**39*(5), 788–793. 27006513 10.2337/dc15-1807

[CR7] Chien, Y. L., Wu, C. S., Chang, Y. C., Cheong, M. L., Yao, T. C., & Tsai, H. J. (2022). Associations between parental psychiatric disorders and autism spectrum disorder in the offspring. *Autism Research,**15*(12), 2409–2419. 36250255 10.1002/aur.2835

[CR8] Daniels, J. L., Forssen, U., Hultman, C. M., Cnattingius, S., Savitz, D. A., Feychting, M., & Sparen, P. (2008). Parental psychiatric disorders associated with autism spectrum disorders in the offspring. *Pediatrics,**121*(5), e1357-1362. 18450879 10.1542/peds.2007-2296

[CR9] Davis, E., Fennoy, I., Laraque, D., Kanem, N., Brown, G., & Mitchell, J. (1992). Autism and developmental abnormalities in children with perinatal cocaine exposure. *Journal of the National Medical Association,**84*(4), 315–319. 1380564 PMC2637680

[CR10] Durkin, M. S., Maenner, M. J., Baio, J., Christensen, D., Daniels, J., Fitzgerald, R., Imm, P., Lee, L. C., Schieve, L. A., Van Naarden Braun, K., Wingate, M. S., & Yeargin-Allsopp, M. (2017). Autism spectrum disorder among US children (2002–2010): Socioeconomic, racial, and ethnic disparities. *American Journal of Public Health,**107*(11), 1818–1826. 28933930 10.2105/AJPH.2017.304032 PMC5637670

[CR11] Eslami Shahrbabaki, M., Barfehie, D., Mazhari, S., Ahmadi, A., & Shafiee, S. (2022). Comparing cognitive functions in patients with schizophrenia and methamphetamine-induced psychosis with healthy controls. *Addiction and Health,**14*(4), 239–243. 37559792 10.34172/ahj.2022.1143 PMC10408749

[CR12] Feng-lei Zhu, Y. J., Wang, L., Zhu, H.-l, Min, X., Ji, Y., & Zou, X. (2023). Delay of diagnosis in autism spectrum disorder and its influencing factors. *Research Square*. 10.21203/rs.3.rs-3193389/v1

[CR13] Goodman, S. H. (1987). Emory university project on children of disturbed parents. *Schizophrenia Bulletin,**13*(3), 411–423. 3629197 10.1093/schbul/13.3.411

[CR14] Gunnarsdottir, E. D., Hallgren, J., Hultman, C. M., McNeil, T. F., Crisby, M., & Sandin, S. (2018). Risk of neurological, eye and ear disease in offspring to parents with schizophrenia or depression compared with offspring to healthy parents. *Psychological Medicine,**48*(16), 2710–2716. 29669615 10.1017/S0033291718000338

[CR15] Healy, S. J., Lewin, J., Butler, S., Vaillancourt, K., & Seth-Smith, F. (2016). Affect recognition and the quality of mother-infant interaction: Understanding parenting difficulties in mothers with schizophrenia. *Archives of Women’s Mental Health,**19*(1), 113–124. 25902956 10.1007/s00737-015-0530-3

[CR16] Kahng, S. K., Oyserman, D., Bybee, D., & Mowbray, C. (2008). Mothers with serious mental illness: When symptoms decline does parenting improve? *Journal of Family Psychology,**22*(1), 162–166. 18266543 10.1037/0893-3200.22.1.162

[CR17] Karunakaran, S., Menon, R. N., Nair, S. S., Santhakumar, S., Nair, M., & Sundaram, S. (2020). Clinical and genetic profile of Autism Spectrum Disorder-Epilepsy (ASD-E) phenotype: two sides of the same coin! *Clinical EEG and Neuroscience,**51*(6), 390–398. 32114799 10.1177/1550059420909673

[CR18] Leng, L. L., Zhu, Y. W., & Zhou, L. G. (2023). Explaining differences in autism detection timing: Age of diagnosis and associated individual and socio-familial factors in Chinese children. *Autism,**28*(4), 896–907. 37491952 10.1177/13623613231187184

[CR19] Levy, S. E., Mandell, D. S., & Schultz, R. T. (2009). Autism. *Lancet,**374*(9701), 1627–1638. 19819542 10.1016/S0140-6736(09)61376-3 PMC2863325

[CR20] Li, Y. A., Chen, Z. J., Li, X. D., Gu, M. H., Xia, N., Gong, C., Zhou, Z. W., Yasin, G., Xie, H. Y., Wei, X. P., Liu, Y. L., Han, X. H., Lu, M., Xu, J., & Huang, X. L. (2022). Epidemiology of autism spectrum disorders: Global burden of disease 2019 and bibliometric analysis of risk factors. *Frontiers in Pediatrics,**10*, 972809. 36545666 10.3389/fped.2022.972809 PMC9760802

[CR21] Liu, C. Y., Hung, Y. T., Chuang, Y. L., Chen, Y. J., Weng, W. S., & Liu, J. S. (2006). Incorporating development stratification of Taiwan townships into sampling design of large scale health interview survey. *J Health Management (chin),**4*, 1–22.

[CR22] Lord, C., Elsabbagh, M., Baird, G., & Veenstra-Vanderweele, J. (2018). Autism spectrum disorder. *Lancet,**392*(10146), 508–520. 30078460 10.1016/S0140-6736(18)31129-2 PMC7398158

[CR23] Oyserman, D., Mowbray, C. T., Meares, P. A., & Firminger, K. B. (2000). Parenting among mothers with a serious mental illness. *American Journal of Orthopsychiatry,**70*(3), 296–315. 10953777 10.1037/h0087733

[CR24] Picardi, A., Gigantesco, A., Tarolla, E., Stoppioni, V., Cerbo, R., Cremonte, M., Alessandri, G., Lega, I., & Nardocci, F. (2018). Parental burden and its correlates in families of children with autism spectrum disorder: A multicentre study with two comparison groups. *Clinical Practice and Epidemiology in Mental Health,**14*, 143–176. 30158998 10.2174/1745017901814010143 PMC6080067

[CR25] Rabha, A., Padhy, S. K., & Grover, S. (2021). Parenting skills of patients with chronic schizophrenia. *Indian J Psychiatry,**63*(1), 58–65. 34083821 10.4103/psychiatry.IndianJPsychiatry_107_20 PMC8106422

[CR26] Ramey, T., & Regier, P. S. (2019). Cognitive impairment in substance use disorders. *CNS Spectrums,**24*(1), 102–113. 30591083 10.1017/S1092852918001426 PMC6599555

[CR27] Riordan, D., Appleby, L., & Faragher, B. (1999). Mother-infant interaction in post-partum women with schizophrenia and affective disorders. *Psychological Medicine,**29*(4), 991–995. 10473327 10.1017/s0033291798007727

[CR28] Shayestehfar, M., Nakhostin-Ansari, A., Memari, A., Hosseini Asl, S. H., & Faghihi, F. (2023). Risk of autism spectrum disorder in offspring with parental schizophrenia: A systematic review and meta-analysis. *Nordic Journal of Psychiatry,**77*(2), 127–136. 35507890 10.1080/08039488.2022.2070664

[CR29] Sipsock, D., Tokadjian, H., Righi, G., Morrow, E. M., Sheinkopf, S. J., R Rhode Island Consortium for Autism and Treatment. (2021). Autism severity aggregates with family psychiatric history in a community-based autism sample. *Autism Research,**14*(12), 2524–2532. 34652072 10.1002/aur.2625 PMC8665120

[CR30] Solmi, M., Song, M., Yon, D. K., Lee, S. W., Fombonne, E., Kim, M. S., Park, S., Lee, M. H., Hwang, J., Keller, R., Koyanagi, A., Jacob, L., Dragioti, E., Smith, L., Correll, C. U., Fusar-Poli, P., Croatto, G., Carvalho, A. F., Oh, J. W., & Cortese, S. (2022). Incidence, prevalence, and global burden of autism spectrum disorder from 1990 to 2019 across 204 countries. *Molecular Psychiatry,**27*(10), 4172–4180. 35768640 10.1038/s41380-022-01630-7

[CR31] Talantseva, O. I., Romanova, R. S., Shurdova, E. M., Dolgorukova, T. A., Sologub, P. S., Titova, O. S., Kleeva, D. F., & Grigorenko, E. L. (2023). The global prevalence of autism spectrum disorder: A three-level meta-analysis. *Front Psychiatry,**14*, 1071181. 36846240 10.3389/fpsyt.2023.1071181 PMC9947250

[CR32] van’t Hof, M., Tisseur, C., van Berckelear-Onnes, I., van Nieuwenhuyzen, A., Daniels, A. M., Deen, M., Hoek, H. W., & Ester, W. A. (2021). Age at autism spectrum disorder diagnosis: A systematic review and meta-analysis from 2012 to 2019. *Autism,**25*(4), 862–873. 33213190 10.1177/1362361320971107

[CR33] Wang, H. E., Cheng, C. M., Bai, Y. M., Hsu, J. W., Huang, K. L., Su, T. P., Tsai, S. J., Li, C. T., Chen, T. J., Leventhal, B. L., & Chen, M. H. (2022). Familial coaggregation of major psychiatric disorders in first-degree relatives of individuals with autism spectrum disorder: A nationwide population-based study. *Psychological Medicine,**52*(8), 1437–1447. 32914742 10.1017/S0033291720003207

[CR34] Wei, H. T., Hsu, J. W., Huang, K. L., Bai, Y. M., Su, T. P., Li, C. T., Lin, W. C., Tsai, S. J., Pan, T. L., Chen, T. J., & Chen, M. H. (2021). Timing of the diagnoses of attention deficit hyperactivity disorder and autism spectrum disorder in Taiwan. *Journal of Autism and Developmental Disorders,**51*(3), 790–797. 29982895 10.1007/s10803-018-3655-1

[CR35] Zhang, B., Wang, H. E., Bai, Y. M., Tsai, S. J., Su, T. P., Chen, T. J., Wang, Y. P., & Chen, M. H. (2021). Inflammatory bowel disease is associated with higher dementia risk: A nationwide longitudinal study. *Gut,**70*(1), 85–91. 32576641 10.1136/gutjnl-2020-320789

[CR36] Zhu, Y., Womer, F. Y., Leng, H., Chang, M., Yin, Z., Wei, Y., Zhou, Q., Fu, S., Deng, X., Lv, J., Song, Y., Ma, Y., Sun, X., Bao, J., Wei, S., Jiang, X., Tan, S., Tang, Y., & Wang, F. (2019). The relationship between cognitive dysfunction and symptom dimensions across schizophrenia, bipolar disorder, and major depressive disorder. *Front Psychiatry,**10*, 253. 31105603 10.3389/fpsyt.2019.00253 PMC6498739

